# Exploring Biochemical Characteristics of Pediatric Hyperdiploid Acute Lymphoblastic Leukemia by Raman Spectroscopy

**DOI:** 10.1021/acs.analchem.5c00410

**Published:** 2025-05-09

**Authors:** Anna M. Nowakowska, Patrycja Leszczenko, Agata Pastorczak, Zuzanna Urbańska, Justyna Jakubowska, Marta Ząbczyńska, Wojciech Mlynarski, Malgorzata Baranska, Kinga Ostrowska, Katarzyna Majzner

**Affiliations:** † Faculty of Chemistry, 37799 Jagiellonian University in Krakow, Gronostajowa 2, 30-387 Krakow, Poland; ‡ Doctoral School of Exact and Natural Sciences, Jagiellonian University in Krakow, Lojasiewicza 11, 30-348 Krakow, Poland; § Department of Pediatrics, Oncology and Hematology, 37808 Medical University of Lodz, Czechoslowacka 4, 92-216 Lodz, Poland; ∥ Department of Genetic Predisposition to Cancer, Medical University of Lodz, Czechoslowacka 4, 92-216 Lodz, Poland

## Abstract

*Hyperdiploid* (HD) B-cell acute lymphoblastic leukemia (ALL) is widely recognized as the most common molecular subtype of leukemia, characterized by the presence of supernumerary chromosomes in the leukemic karyotype. While HD B-ALL is often associated with a favorable prognosis, an important subset of patients still experience relapse, reflecting the biological heterogeneity of this subtype. Current genomic and epigenetic research has shed light on the molecular complexity of HD B-ALL, yet rapid methods for capturing both the metabolic state and the chromosomal content of individual cells remain limited. Here, we introduce a novel Raman spectroscopy (RS)-based approach for the single-cell analysis of HD B-ALL. By detecting characteristic spectroscopic signatures of nucleic acids, proteins, and lipids, RS not only distinguishes malignant cells from normal B cells, but also discriminates between HD B-ALL and other molecular subtypes, including *TCF3-PBX1*, *KMT2A-r*, *BCR-ABL1*, and *TEL-AML1*. Notably, we developed a partial least-squares regression (PLS-R) model capable of accurately predicting chromosome number from each cell’s Raman spectrum, thereby linking molecular fingerprints directly to genomic aberrations. This integrative spectroscopic strategy captures disease heterogeneity and informs therapeutic strategies. Taken together, our proof-of-concept findings highlight RS as a powerful, noninvasive tool for quantifying chromosomal alterations and metabolic phenotypes, adding crucial insights into the complex biology of HD B-ALL and paving the way for broader applications in precision medicine.

## Graphical abstract

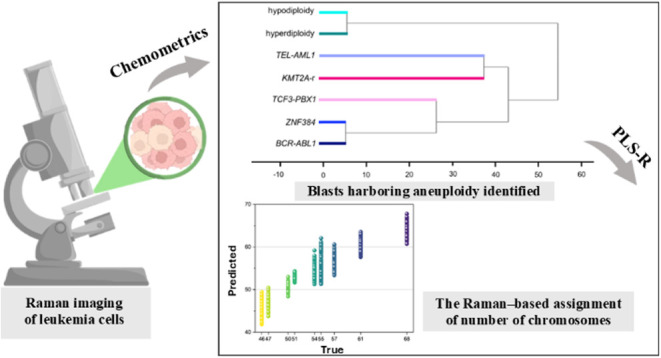

## Introduction

Precursor B-cell acute lymphoblastic leukemia (BCP-ALL) is hallmarked by chromosomal abnormalities, including rearrangements and aneuploidies.^1^ Among aneuploidies, *hyperdiploidy* (HD) is the most prevalent subtype of childhood acute B-cell leukemia, characterized by the presence of more than 46 chromosomes in a leukemic karyotype.^2–5^ Childhood HD ALL is associated with a favorable prognosis.^2,6^ HD leukemias originate from individual immature B-cell precursor blasts that are transformed at an early stage during fetal development.^7^ The median age of children diagnosed with HD ALL is ∼4 years. Clinical outcome of HD leukemia,^5,8,9^ to some extent, depends on the combination of additional chromosomes, with trisomies of chromosomes 5, 9, 10, and 18 being predictors of relapse, while trisomies of chromosomes 4, 10, and 17 are markers of outstanding prognostic benefit.^10^ Moreover, the co-occurrence of specific microdeletions, i.e., *IKZF1*, *CDKN2A*, *CDKN2B*, or point mutations in genes such as *CREBBP* or *KRAS*, can also affect the prognosis.^3,11,12^ Primarily, HD was divided into two types: low (47–50 chromosomes) and high (>50 chromosomes).^13^ However, the latest studies have provided a more complex classification that defines five subtypes of HD, i.e., (i) classical, (ii) nonclassical, (iii) near-triploid, and biclonal types: (iv) biclonal hyperhaploid and HD, and (v) biclonal hypodiploid and near-triploid. This reveals the complex nature of the HD form of ALL. Therefore, the precise mechanism involved in the generation of HD or its role in leukemogenesis remains elusive and requires further investigation.^12,14^ The high-HD (HHD) karyotype, characterized by 51–65 chromosomes, is found in ∼30% of BCP-ALL cases, resulting in an overall favorable prognosis.^15^ The low-HD karyotype, defined by the presence of 47–50 chromosomes, is associated with an intermediate prognosis and worse patient outcomes as compared to HHD ALL.^12,16^

Currently, childhood HD ALL is routinely diagnosed based on classical and molecular karyotyping, DNA content measurements, and fluorescence in situ hybridization (FISH) using centromere probes.^17^ However, in some cases, ulterior genetic alterations are undetectable by standard cytogenetic methods (e.g., FISH or G-banding).^12^ To overcome these limitations, a microarray approach has been shown as a powerful tool providing genome-wide screening for copy number alterations that elude standard diagnostic protocols.^18^ However, since 25% of patients experience recurrence of the disease, a significant challenge in HD management still concerns a diagnostic approach that allows the identification of patients with a high predisposition to relapse.^2^ As the range of technologies supporting and complementing existing diagnostic tools constantly evolves, assessing new, detailed information on genomic peculiarities, improved diagnostics, and risk stratification seems achievable in perspective. Among them are techniques based on Raman spectroscopy (RS).

The potential of RS has been presented in the analysis of the rich biochemical and metabolic tapestry of leukemic cells,^19–21^ as well as in the diagnosis of leukemia.^22–26^ By taking advantage of light-and-matter interactions, specifically Raman scattering, RS provides detailed insight into the unique molecular fingerprints of blood cells in a noninvasive and sensitive manner.^23^ Currently, subcellular analysis is based primarily on fluorescence spectroscopy and selected dyes that bind specifically to the molecules of interest.^17^ However, because of the wide emission bands of such fluorescent dyes, there are limits to the number of fluorescent labels that can be used simultaneously.^27^ RS does not have this limitation because it provides comprehensive information on the molecular structure of single cells in a label-free manner. Moreover, RS combined with machine learning-based data analysis methods is becoming a powerful tool in diagnosing blood diseases at the cellular and even subcellular level.^19–21^

Taking into account the emerging need to study unique metabolic characteristics of specific molecular subtypes of leukemia, we hypothesize that RS can be used for the identification of B-ALL cells with HD and their differentiation from normal B cells and other selected molecular subtypes of leukemia (*hypodiploidy, TCF3-PBX1, KMT2A-r, BCR-ABL1, ZNF384*, and *TEL-AML1*). Additionally, we investigated how the molecular composition of leukemic cells is influenced by chromosome number and explored how Raman spectroscopy can be used to detect these changes.

## Materials and Methods

### Preparation of Clinical Samples

Mononuclear cells were isolated from bone marrow samples using density gradient centrifugation with Histopaque-1077 (Sigma-Aldrich, Saint Louis, MO) and collected at the time of ALL diagnosis from pediatric patients (*n* = 16) included in the study. HD ALL cases were identified based on classical karyotyping of leukemic cells and using microarray testing. A detailed protocol for sample preparation was presented elsewhere.^28^

### Microarray Analysis

Gene copy number aberrations (CNAs) were analyzed using Cytoscan HD microarrays (Applied Biosystems, Thermo Fisher Scientific, Waltham, MA) that comprise 2,670,000 markers, including 750,000 single nucleotide polymorphisms (SNP) and 1,900,000 nonpolymorphic copy number variation probes (CNV). An assay was conducted with the input of 250 ng of genomic DNA isolated from leukemic cells, which was processed according to the manufacturer’s current protocol. This protocol involved digestion with the NspI enzyme, PCR amplification, ligation with restriction fragment-linked adapters, purification of PCR products using magnetic beads, fragmentation with DNase I, and labeling with terminal deoxynucleotidyltransferase (TdT). The samples were then hybridized overnight (16–18 h) in a 49-format array. After incubation, the arrays were washed and stained on the GeneChip Fluidics Station 450 and then scanned with a GeneChip Scanner 3000. The system generated CEL files containing the signal intensities of the probes, which were then converted using Chromosome Analysis Suite v 4.5 software (ChAS, Thermo Fisher Scientific, Waltham, MA) to CYCHP files containing a copy number, loss of heterozygosity (LOH), and mosaicism information. The analysis utilized a panel of 1,286 leukemic genes, applying filters for the detection of copy number alterations (CNAs): 50 probes for duplications and 20 probes for deletions. The loss of heterozygosity was reported if the region was covered by a minimum of 50 probes and exceeded 3000 kb in length.

### Raman Imaging of Cells

Raman imaging of single cells was performed using a confocal Raman microscope (WITec α 300, WITec GmbH, Ulm, Germany) equipped with an air-cooled 532 nm laser, a CCD detector (Andor Technology Ltd., Belfast, Northern Ireland), and a 600 grooves/mm grating (BLZ = 500 nm) with a spectral resolution of approximately 3 cm^–1^. The measurement protocol was described in detail in ref 28. A total of 250–500 μL of cell suspension, deposited on CaF_2_ windows (Crystran LTD, Poole, U.K.), was measured by illumination with a 63× water immersion objective (NA = 1, Zeiss W Plan-Apochromat 63×, Oberkochen, Germany). Raman imaging was performed by using a sampling density of 1 μm and an exposure time of 0.5 s *per* spectrum. Measurements were conducted at room temperature, with at least 50 morphologically intact, round cells analyzed *per* sample.

### Data Preprocessing and Analysis

The initial data preprocessing was performed using Project FIVE 5.1 Plus software (WITec GmbH, Ulm, Germany). Spectral preprocessing included the removal of artifacts from cosmic radiation (cosmic ray removal, filter size: 3, and dynamic factor: 8), the subtraction of background contributions, and residual autofluorescence (polynomial fitting, third order). The *k*-means cluster analysis (KMCA) was performed by using the Manhattan distance calculation to separate cell spectra, background spectra, and single organelle spectra for each measured cell. This approach enabled the grouping of single Raman spectra into classes based on their spectral similarities, which are directly related to their biochemical characteristics, and the extraction of mean spectra of cells and their major structural components, such as the nucleus and cytoplasm.

Further analysis, such as principal component analysis (PCA) or orthogonal partial least-squares regression (O-PLS-R), was carried out on the averaged spectra of single cells using Solo+Mia 9.1 software (eigenvector Research, Wenatchee, WA). Spectral analyses were performed in the fingerprint region (i.e., 1800–500 cm^–1^). All single-cell averaged spectra were smoothed using a Savitzky–Golay filter (third-order polynomial, 13 points) and then subjected to multiplicative scattering correction and mean centering. Venetian blind cross-validation was applied to construct the models. Approximately 85% of the data was used to build the regression model, and the remaining 15% was used to test the model, excluding the data set used during training. The final data presentation was obtained using OriginPro 2022 (OriginLab, Northampton, MA).

## Results and Discussion

### Differentiation of HD B-ALL Blasts from Normal B Cells

The first step in the diagnostic algorithm for any disease is to identify abnormal cells and distinguish them from their healthy counterparts. In our previous studies, we showed that leukemic cells representing various subtypes (including T-ALL^19^ and B-ALL^29^ cells with *BCR-ABL1*, *TCF-PBX1*, *TEL-AML1*,^20^ and *KMT2A*-r fusion genes^21^) can be distinguished from normal lymphocytes based on (i) the intensity of marker bands associated with nucleic acids that characterize B lymphocytes and (ii) bands derived from protein and lipid vibrations that were specific for leukemic cells. In this study, we examined the HD ALL subtype, a highly diverse variant linked to an increased risk of relapse. This prompted us to explore this subtype using RS, comparing it with other previously characterized subtypes (*TCF3-PBX1, KMT2A-r, BCR-ABL1, and TEL-AML1*), as well as normal blood cells, which may be challenging due to the high intensity of signals related to nucleic acids in normal B cells and HD ALL. Here, we compared the Raman profiles of both cell types using an unsupervised chemometric method, i.e., PCA. A total of 397 spectra of B lymphocytes from 8 healthy donors and 381 spectra of HD ALL blasts collected from 16 pediatric patients were used for comparison (Figure 1). The score plot (Figure 1a) shows satisfactory separation between the spectra of normal B lymphocytes (marked in purple) and the Raman profiles of HD ALL (marked in aqua). Separation is observed along the first two principal components (PC-1 and PC-2), which represent 27 and 11% of the data variability, respectively. Most of the spectra of HD ALL cells are positioned on PC-1­(+) and B cells on PC-1(−), whereas the spectra of normal (PC-2­(+)) and malignant (PC-2(−)) cells were mainly separated along PC-2.

**Figure 1:**
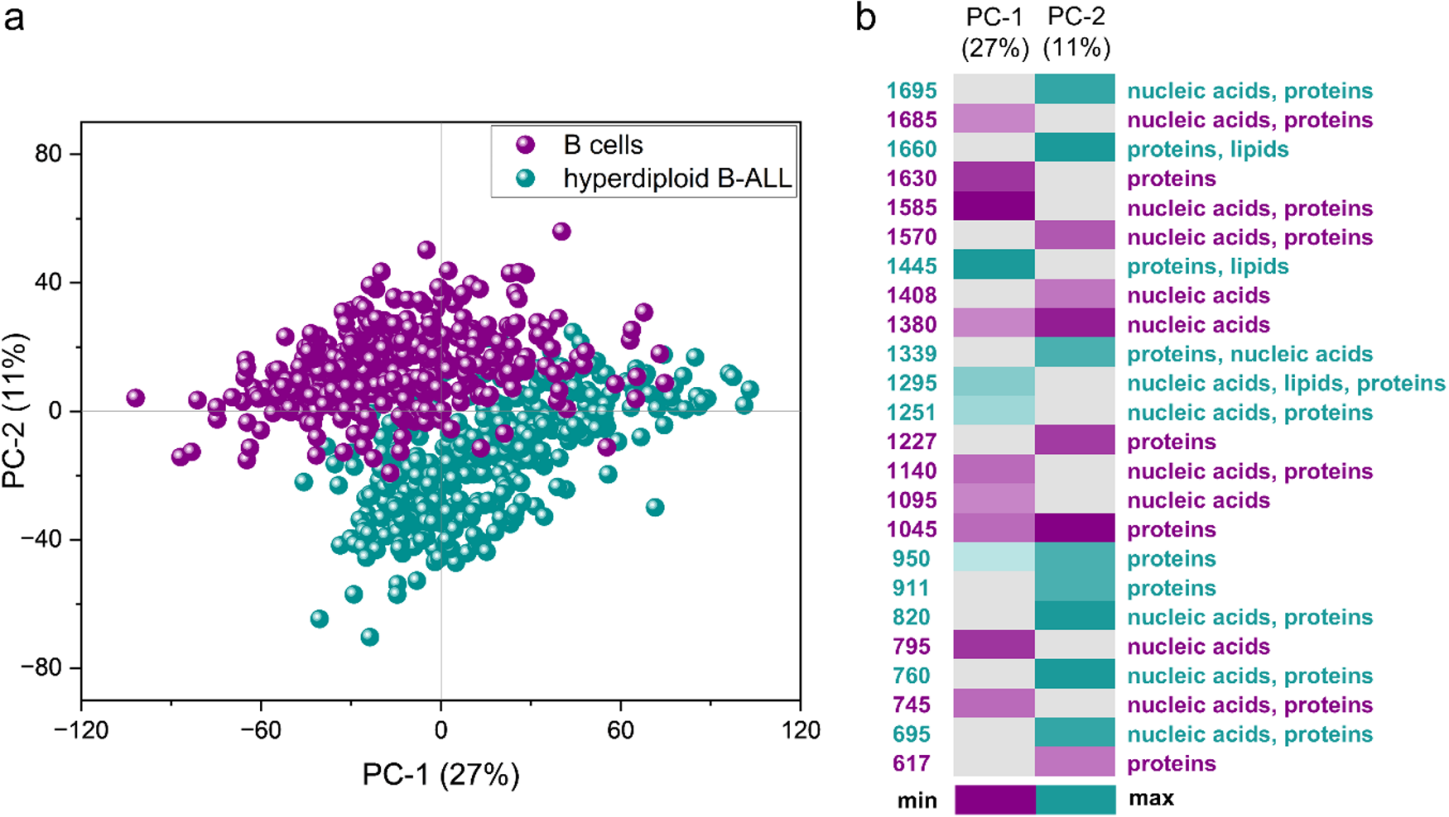
Comparison of the spectra of normal B cells (purple, *n*_p_ = 8, *n*_s_ = 397) with HD B-ALL lymphoblasts (aqua, *n*_p_ = 16, *n*_s_ = 381). (a) Score plot of PCA along the first two PCs (PC-1 and PC-2). (b) Loading plots of PC-1 and PC-2 are presented in a color scale. Only bands for which the PC-1 and PC-2 had the highest values (PC-1: >0.05 and <−0.05, PC-2: >0.07 and <−0.07) were included. The PC-2 loading values were multiplied by (−1) to maintain the color scale. PCA analysis was performed in the spectral range of 1800–600 cm^–1^.

Analyzing the PC-2 loading plot (Figure 1b), it can be concluded that B lymphocytes and HD ALL cells differ, among others, in the protein composition, as evidenced by the bands at 1630, 1227, 1045, and 617 cm^–1^ (normal B cells) and at 1660, 1251, 950, 760, and 695 cm^–1^ (HD ALL cells). Furthermore, B cells were characterized by the bands originating primarily from nucleic acids, i.e., 1685, 1585, 1570, 1380, 1140, 1095, 795, and 745 cm^–1^. This suggests an increased proportion of the nucleus relative to the size of the entire cell or a different degree of chromatin condensation in healthy B cells compared to HD leukemic cells. This is somewhat surprising as HD cells have extra chromosomes in their karyotype. However, our current and previous studies^19–21^ confirmed that normal B lymphocytes are hallmarked by increased intensities of bands assigned to DNA and RNA,^30^ which is probably related to the morphology of lymphocytes, which have a large nucleus.^31,32^ Furthermore, in accordance with our previous studies, leukemic cells are characterized by an elevated protein and lipid content, as evidenced by the higher intensity of the bands at 1445 cm^–1^ (CH_2_/CH_3_ plane bending) and 1295 cm^–1^ (CH deformation vibration). Elevated levels of lipids and proteins in cancer cells may be associated with higher metabolic activity in malignant cells compared to normal blood cells.^24,33–35^ Spectroscopic characterization of HD cells also includes the band at 1339 cm^–1^ (CH_2_/CH_3_ fan-shaped, bending, twisting) and at 911 cm^–1^, which may correspond to amino acids such as proline and ribose in RNA. Again, the Raman bands characteristic of leukemic cells originate mainly from protein–lipid components, and the marker bands for B cells are related to vibrations that can be assigned to nucleic acids. It appears to be a universal spectroscopic fingerprint of the metabolism of blood cells upon neoplastic transformation.

### Raman Image of HD B-ALL Cells Compared to Other Subtypes of B-ALL

Malignant and normal lymphocytes exhibit expected differences in metabolism, a notion that has been repeatedly confirmed by RS using various analytical approaches.^22–26^ However, as previously described,^19–21^ defining differences between genetic subtypes of the same disease is challenging. The Raman spectra of HD ALL cells were subjected to PCA in comparison with several other subtypes of B-ALL (Figure 2). We ensured that the spectral sets studied were balanced using approximately 100–170 mean cell spectra of each B-ALL subtype collected from samples from different patients (the number of patients for each subtype was indicated as *n*_p_ and the number of spectra for each subtype by *n*_s_): *TCF3-PBX1* (light pink, *n*_p_ = 12, *n* = 175), *KMT2A-r* (dark pink, *n*_p_ = 12, *n*_s_ = 176), *BCR-ABL1* (navy, *n*_p_ = 11, *n*_s_ = 139), *ZNF384* (blue, *n*_p_ = 7, *n*_s_ = 96), and *TEL-AML1* (light blue, *n*_p_ = 13, *n*_s_ = 162). On the contrary, 619 mean cell spectra from HD ALL cells were added for comparison (aqua, *n*_p_ = 13). A satisfactory division was obtained (Figure 2a). The spectra of HD B-ALL were divided along PC-2, which describes 11% of the total variability, substantially less than that in the comparison of HD leukemic cells and B lymphocytes. This observation indicates a greater spectral similarity between the different leukemia subtypes than that for normal lymphocytes. It is worth noting that in this analysis, three HD samples were excluded from the PCA because their cell spectra differed the most from those of other cells within the same subtype. That was motivated by the desire to capture the general picture of the differences that characterize most HD lymphoblasts in the background of other molecular subtypes of B-ALL. Based on the distribution of the spectra of cells with other genetic abnormalities than HD (Figure 2a), we see that they are mixed, with one exception for the *KMT2A* rearrangement, whose spectral characterization was described in detail in ref 21. In the case of *KMT2A*-r, its most distinguishing spectral characteristics are related to the different protein conformations compared to those of other subtypes. As shown in Figure 2b, the spectra of HD are characterized by bands that can be assigned primarily to proteins, i.e., at 1700 cm^–1^ (carbonyl vibrations in amino acids—Asp, Glu), 1675 cm^–1^ (amide I), 1600 cm^–1^ (Phe), 1399 cm^–1^ (CH_2_ deformation vibration), and 1337 cm^–1^ (CH_2_/CH_3_ fan, bending, twisting). The band at 1130 cm^–1^ observed in the spectra of HD B-ALL cells may be a marker for RNA or proteins, especially porphyrin systems, similar to the 756 cm^–1^ band. The bands, which can be associated with vibrations of cytochrome proteins in HD cells, may indicate higher metabolic activity compared with other subtypes, including *TCF3-PBX1*, *KMT2A-r*, *BCR-ABL1*, *ZNF384*, and *TEL-AML1*. Additionally, the band at 960 cm^–1^, observed in HD cells, can be assigned to the vibrational modes of the phosphate residues. The remaining ALL subtypes are characterized by distinct bands, which can also be assigned to specific proteins, such as the bands at 1630 cm^–1^ (amide I), 1570 cm^–1^ (Phe and Trp), 990 cm^–1^ (one of the β-sheet marker bands), and 660 cm^–1^ (Phe and Tyr). Additionally, the band at 1440 cm^–1^ (CH_2_/CH_3_ plane bending) observed in the spectra of B-ALL blasts, excluding HD cells, can be attributed to proteins and lipids. Interestingly, the band related to nucleic acids at 790 cm^–1^ appears on the side of the PC-2 loading, not characterizing the HD B-ALL subtype. This is surprising as HD cells exhibit an increased number of chromosomes in the nucleus, which would lead to a higher intensity of Raman signals from nucleic acids. Furthermore, higher values of the DNA index (DI) in HD cells (DI between 1.16 and 1.6) compared to control cells (DI of 1.0) are associated with a higher content of genetic material in HD ALL, allowing for efficient stratification of patients and identification of this subtype.^36–39^ However, the results showed that RS provides holistic information on cell metabolism, indicating Raman features derived from proteins, including cytochromes, and based on these features, dissects HD from other types of BCP-ALL. Our findings suggest that HD B-ALL represents a unique and metabolically distinct group when compared to other subtypes.^10,40,41^

**Figure 2:**
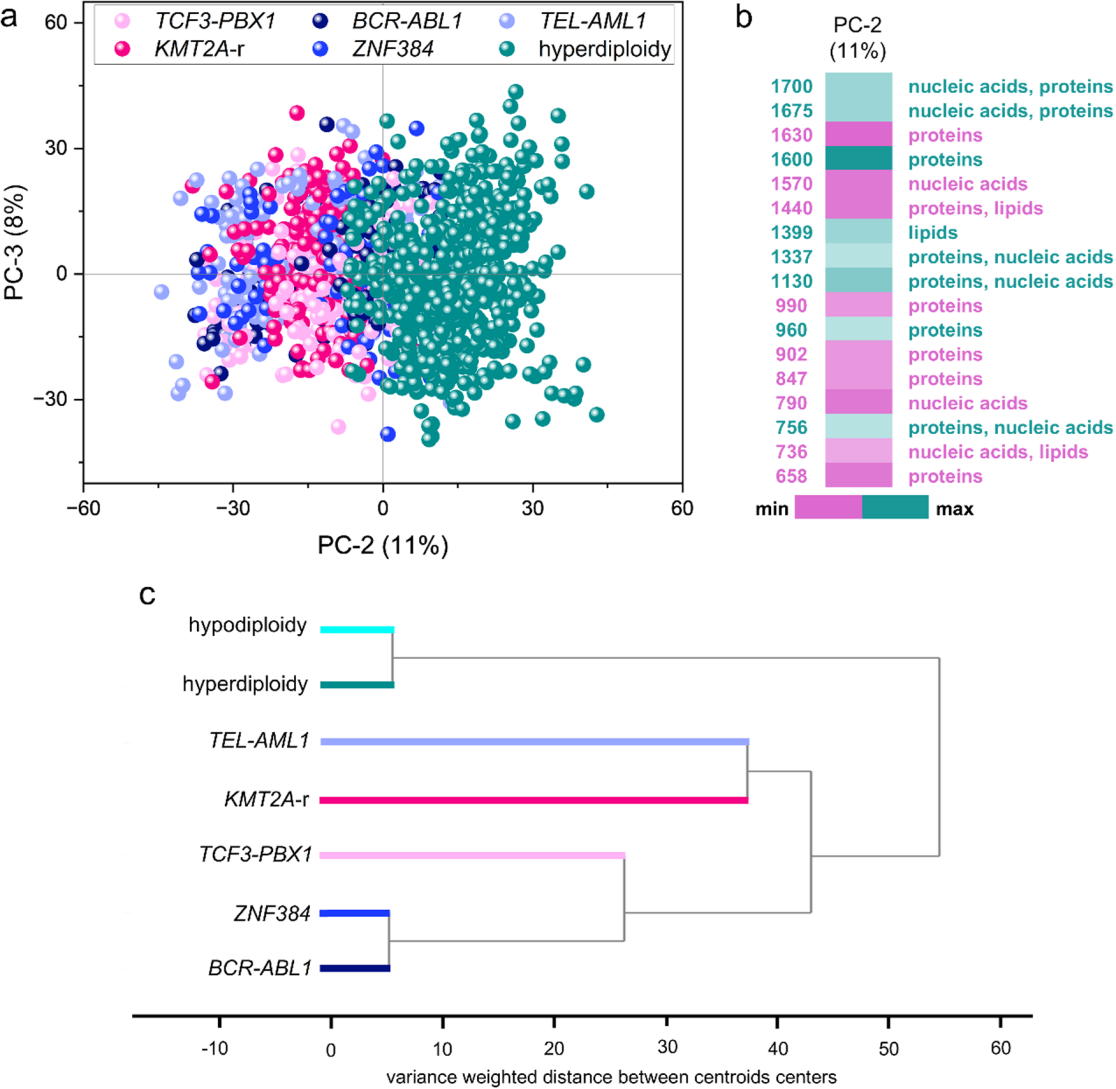
Comparison of spectra of *HD* lymphoblasts (marked in aqua) and a mixture of other subtypes of B-ALL studied: *TCF3-PBX1* (marked in light pink, *n*_p_ = 12, *n*_s_ = 175), *KMT2A*-r (dark pink, *n*_p_ = 12, *n*_s_ = 176), *BCR-ABL1* (navy, *n*_p_ = 11, *n*_s_ = 139), *ZNF384* (blue, *n*_p_ = 7, *n*_s_ = 96), and *TEL-AML1* (light blue, *n*_p_ = 13, *n*_s_ = 162). (a) Score plot of principal component factors PC-2 and PC-3. (b) Loading plot for the PC-2 component presented on a color scale. Only bands for which the PC-2 loading had the highest values (greater than 0.06 and less than −0.06) were included. PCA analysis was performed in the spectral range of 1800–600 cm^–1^. (c) Hierarchical cluster analysis of the average spectra of the studied subtypes.

Figure 2c presents the results of the hierarchical cluster analysis performed in the fingerprint range on the mean spectra of the molecular subtypes of BCP-ALL. Average spectra were calculated from all available mean cellular spectra of a given ALL subtype. The colors associated with the individual subtypes were added to facilitate the analysis. It can be stated that the most distinctive groups are those harboring aneuploidy, *HD*, and *hypodiploidy*, respectively. Both subtypes are more similar to each other than the different molecular entities of ALL. Nevertheless, it can be assumed that lymphoblasts with increased chromosomes constitute the most distinct group among the genetic subtypes studied.

The results presented above confirm a distinct biochemical specificity of HD ALL despite the relatively high heterogeneity of this subtype, as shown in the PCA score plot in Figure S1. Evidence of the high biochemical and metabolic variability of HD ALL cells, at both the single-cell level and the patient level, is directly evident in the entire Raman profile. One of the sources of variability in HD ALL cells might also be related to co-occurring genetic alterations, which directly affect the biochemical processes within the leukemic cells, reflected in the positions and intensities of the Raman features.^11,12^

### Molecular Composition of HD B-ALL Cells Depends on the Number of Chromosomes

As discussed above, the Raman fingerprint of HD ALL lymphoblasts is not directly related to the nucleic acid content but primarily to the protein–lipid composition. However, to some extent, Raman signals from HD cells depend on the number of chromosomes in lymphoblasts. To better understand the molecular variability of HD cells and verify the hypothesis that the spectral profile of these cells is related to the number of chromosomes, we developed an O-PLS-R-based regression model, which directly links the spectral profile of the cells with the total number of chromosomes in malignant cells (Figure 3).

**Figure 3:**
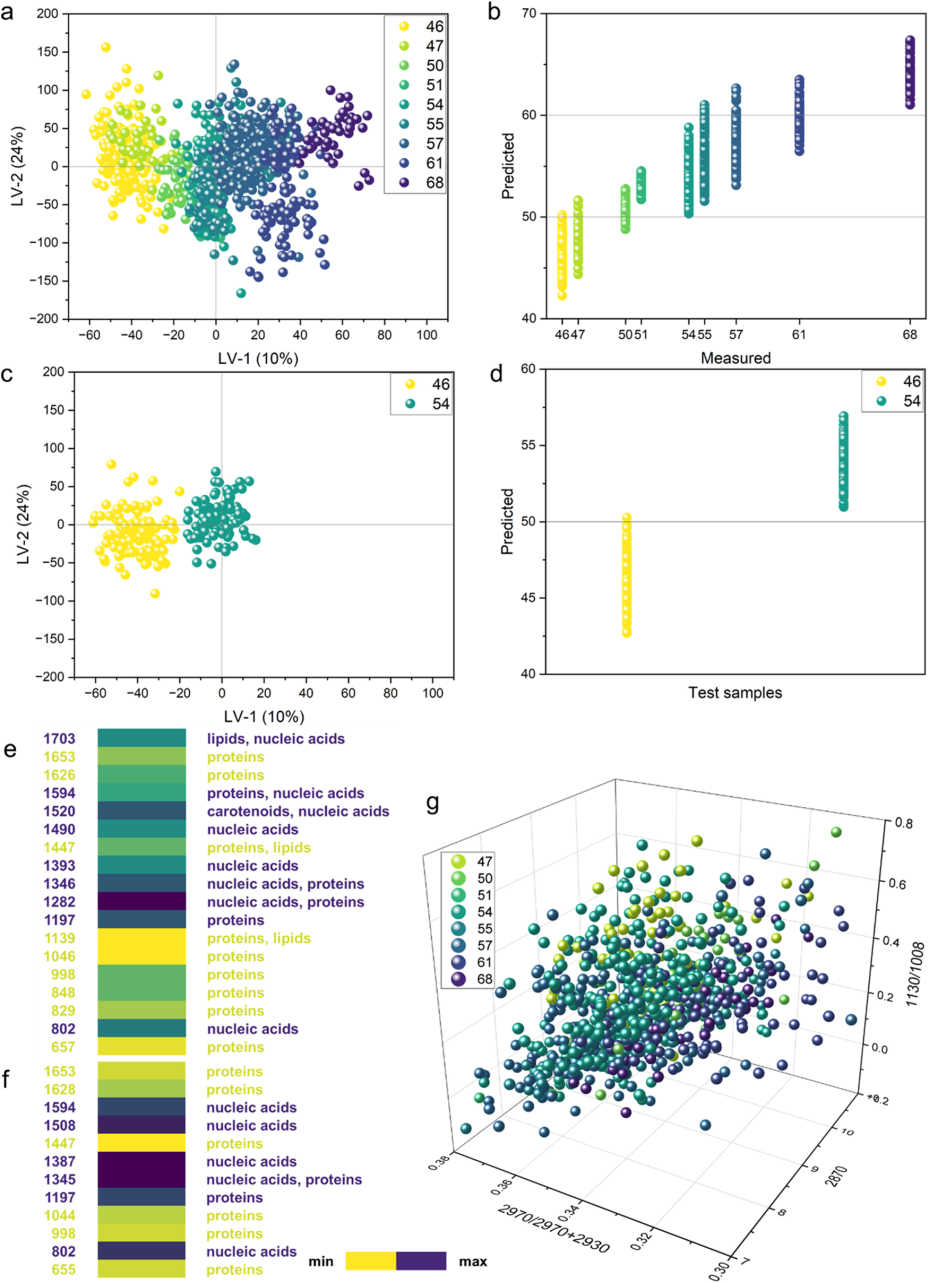
O-PLS-R model that examines the relationship between the Raman signal and the number of chromosomes in clinical samples calculated on the whole-cell spectra. (a) Score plot of latent variables LV-1 and LV-2 for the training data set. In total, six LVs were used. (b) Model calibration result. (c) Score plot of the latent variables LV-1 and LV-2 for the test data set, which was not included in model training. (d) Prediction results of the model in test samples. (e) Plot of the regression vector of the model is presented on a color scale. Only bands for which the variable importance in projection (VIP) scores had the highest values (>1) were included. (f) Plot of the LV-1 loading of the model is presented on a color scale. Only bands for which the LV-1 loading had the highest values (greater than 0.06 and less than −0.06) were included. O-PLS-R analysis was performed in the spectral range of 1800–600 cm^–1^. (g) Graphical representation of the integral intensity ratios of selected characteristic bands of whole-cell spectra. The samples were colored according to the number of chromosomes designated according to panel legend (a).

The PLS-R model was constructed on total *n*_s_ = 1241 mean cell spectra collected from *n*_p_ = 16 patients with identified HD. To extend the calibration range for cells containing standard 46 chromosomes, the training set included spectra from five samples carrying other B-ALL subtypes, including the following: *TCF3-PBX1* (*n*_p_ = 1, *n*_s_ = 64) and *TEL-AML1* (*n*_p_ = 2, *n*_s_ = 104), marked in yellow. The algorithm was also trained using a set of HD samples with the following chromosome numbers: 47 (*n*_p_ = 1, *n*_s_ = 56), 51 (*n*_p_ = 1, *n*_s_ = 60), 54 (*n*_p_ = 3, *n*_s_ = 172), 55 (*n*_p_ = 3, *n*_s_ = 151), 57 (*n*_p_ = 3, *n*_s_ = 199), 61 (*n*_p_ = 2, *n*_s_ = 98), and 68 (*n*_p_ = 1, *n*_s_ = 42). Furthermore, to complete the calibration curve, we added one hypodiploid sample (with 50 chromosomes) from a patient (*n*_p_ = 1, and *n*_s_ = 59). Standard hypodiploidy is diagnosed with a chromosome number of less than 45. Still, the sample used for the PLS-R model was unique and contained a largely duplicated single set of 21 chromosomes.

The distribution of the training data set, which contains HD cell spectra in the space of latent variables with respect to LV-1 and LV-2, is shown in Figure 3a. In total, six LVs, altogether describing 69.3% of the variability, were used. Points representing single-cell spectra were color-coded according to the number of chromosomes determined by using standard cytogenetic methods. Regardless of the subtype of leukemia (*HD*, *hipodiploidy*, *BCR-ABL1*, *TCF3-PBX1,* and *TEL-AML1*), a gradient transition depending on the number of chromosomes is observed, highlighted by the color transition from yellow to green (cells with *hipodiploidy*, and *BCR-ABL1*, *TCF3-PBX1, TEL-AML1* mutations, as well as blasts with *low HD*) through shades of aqua (spectra of lymphoblasts with approximately 54 chromosomes) to *high-HD* blue samples (with the number of chromosomes exceeding 60). Additionally, the spectra of HD lymphoblasts were separated from those of cells representing other molecular subtypes, which had a diploid number of chromosomes (equal to 46). Moreover, despite their genetic and biochemical distinctiveness, the spectra of hypodiploid cells were also correctly assigned to the corresponding numbers of chromosomes. The predictions of O-PLS-R model are shown in Figure 3b. The *x*-axis shows the chromosome numbers determined by classical cytogenetic analyses. The *y*-axis indicates the number of chromosomes that the model estimates for each spectrum. For all samples, the predicted values are not discrete but within a range centered around the value determined by the reference method. However, at this stage, the model cannot recognize the number of chromosomes in lymphoblasts deterministically, but it can approximate this value within a certain range. It is visible that the molecular composition of cells strongly depends on the number of chromosomes, which is a predictive factor that allows the identification of leukemia cells, especially those with HD.

A separate data set (not used in the training) was used to validate the model’s performance. It included spectra from three samples with the following chromosome numbers: 46 (sample with the *BCR-ABL1* fusion gene, *n*_p_ = 1, *n*_s_ = 62 and sample with the *TCF3-PBX1* fusion gene, *n*_p_ = 1, *n*_s_ = 71), and HD samples containing 54 (*n*_p_ = 2, *n*_s_ = 100) (representing classical HD). The spectra of these samples were classified using the O-PLS-R model in respective groups, which were color-coded according to the number of chromosomes determined by cytogenetic methods. The results were presented in the space of latent variables (LV-1 and LV-2, Figure 3c) and as a graph showing the number of chromosomes predicted by the model (Figure 3d). The PLS-R model accurately predicted the number of chromosomes, enabling us to confirm its effectiveness.

In Figure 3e, a regression vector of the O-PLS-R model is displayed, along with the loading of LV-1, which indicates Raman features related to the observed biochemical composition that depends on the number of chromosomes in cells. The LV-1 loading, describing 9.89% variability, indicated bands mainly originating from vibrations of nucleic acids (1594, 1508, 1387, 1345, and 802 cm^–1^), which characterized samples with an increasing number of chromosomes (separated along LV-1 and positioned on the positive side of LV-1). The regression vector, which accounts for the overall variability across all LVs, also identified bands that can be attributed to nucleic acids, including the bands at 1594, 1520, 1490, 1393, 1346, 1282, and 802 cm^–1^. Surprisingly, many of the Raman features visible in the regression vector can be assigned to proteins or lipids, e.g., at 1626, 1447, 1139, 1046, 998, 848, 829, or 657 cm^–1^. It seems that along with the change in the number of chromosomes, differences in the composition of protein and lipid content related to metabolic alterations are dominant in the Raman profiles of malignant cells. Our results also show that Raman signals specific to nucleic acids are directly correlated with the number of chromosomes, but this is not a dominant source of variability in leukemic cells. The coefficient of determination (*R* ^2^) for model calibration was equal to 0.87; for cross-validation, it was equal to 0.87; and for prediction, it was 0.86. The values obtained were satisfactory yet still provided room for improvement. Adding more samples during the calibration stage is essential to enhance the model’s performance. This should involve filling in the gaps for previously unrepresented chromosome numbers and including a more significant number of samples from various patients that correspond to specific chromosome counts (significantly above 60). Such an approach would minimize the influence of individual variability and enable a more balanced representation of classes, which is currently a limitation of the presented model. Additionally, including spectra of samples containing more chromosomes (which are currently under-represented) may also increase the proportion of Raman signals derived from nucleic acids in the regression vector or LV-1 loading, thereby enhancing their contribution to the classification of cells with hyperdiploidy. The performance of the model may be impacted by including atypical samples, such as hypodiploidy with 50 chromosomes or triploid leukemia (68 chromosome sample). However, our objective was to obtain an algorithm as robust as possible and verify its usefulness in predicting the number of chromosomes in patient samples, which was successfully presented in this article. We also performed an analysis on the Raman spectra of the nuclei extracted using KMC analysis (Figure S2), and the results were very similar. This is likely due to the fact that the nucleus occupies the predominant area of the cell and nuclear signals make a dominant contribution to the cellular spectrum. As can be seen, the O-PLS-R model correctly estimates the number of chromosomes but not the leukemia subtype, which may be considered to be a limitation. To further enhance the algorithm and make it even more beneficial for the diagnostic procedure, it is worth considering expanding the data set with additional samples designated as outliers, which will more accurately reflect the situations that may occur in daily clinical practice. Particularly interesting from this point of view could be ALL cases harboring a biclonal karyotype with the coexistence of hypodiploid and HD clones that currently can be captured solely by classical cytogenetic methods. In contrast, masked hypodiploid leukemias, which show duplicated hypodiploid clones in the karyotype, cannot be distinguished from high-HD cases by using classical karyotyping. Still, they can be recognized using microarray testing based on the presence of regions of a loss of heterozygosity. However, because hypodiploid ALL cells display distinct biological features associated with cell metabolism, they could be identified using RS.^2,3^

Additionally, to investigate the variability of HD samples, the analysis of marker bands was performed in selected ranges corresponding to the vibrations of proteins and nucleic acids (Figure 3f). The band ratios in the 3030–2800 cm^–1^ region were calculated. The *x*-axis shows the integral intensity values of the 2870 cm^–1^ band (limits: 2910–2830 cm^–1^), which provides information on the lipid content in the sample. On the *y*-axis, the ratio of the integral intensity values of 2970 cm^–1^ (limits: 3000–2956 cm^–1^) to the sum of 2970 and 2930 cm^–1^ (limits: 2956–2910 cm^–1^) was calculated, referring to the nucleic acid content. Additionally, the 1130 cm^–1^ band was selected in the range of 1150–1118 cm^–1^ compared to 1008 cm^–1^ (1025–985 cm^–1^), thus obtaining information on the content of hemoproteins relative to all proteins. Lymphoblasts with high HD (dark blue) seem to have higher contributions from heme proteins. On the other hand, cells with a lower number of additional chromosomes (green) fit in the upper part of the graph.

## Conclusions

Using RS combined with machine learning methods, we demonstrated the unique biological features of the HD subtype of B-ALL. Based on the Raman profiles of single cells, malignant B lymphocytes can be distinguished relatively easily from normal ones. Considering that HD cells exhibit supernumerary chromosomes in their karyotypes, it was somewhat surprising to identify bands assigned to nucleic acids in the spectra of B cells rather than in HD B-ALL cells. However, this observation demonstrated that Raman features related to the nuclei are universal markers that differentiate normal blood cells from their malignant counterparts, as previously shown in our research.^19–21^ Additionally, HD cells were characterized by higher protein–lipid content.

This study aimed to differentiate HD ALL cases from other molecular subtypes of BCP-ALL using RS. We demonstrated that HD B-ALL can be distinguished from other leukemic entities, including *TCF3-PBX1*, *KMT2A-r*, *BCR-ABL1*, and *TEL-AML1*, based on their Raman spectra. The results show a correlation between the number of chromosomes and the Raman spectra of individual cells, which can potentially be used for the spectral evaluation of the complement of chromosomes, not only in clinical samples with HD but also in the case of other leukemia subtypes. Interestingly, the model indicated that the variations in the Raman profile were not solely linked to signals from nucleic acids but primarily related to the intensity of bands corresponding to proteins, with some contribution from lipids. This suggests that RS highlights how aneuploidy-driven shifts in cellular metabolism and chromatin organization can be captured through protein, lipid, and other biochemical signatures, providing a spectral fingerprint directly related to cell activity. As the next step, a developed regression model requires further refinement and validation using a larger sample pool.

## Supplementary Material



## Notes

Results of the PCA analysis of HD samples based on their karyotype (Figure S1); results of the O-PLS-R analysis of leukemic cells according to the number of chromosomes (Figure S2) (PDF)
