# Supplementary material for: Exploring Biochemical Characteristics of Pediatric Hyperdiploid Acute Lymphoblastic Leukemia by Raman Spectroscopy
Source: Anal Chem. 2025 May 9;97(19):10319–27. doi: 10.1021/acs.analchem.5c00410 (PMC12096350; doi:10.1021/acs.analchem.5c00410)
Supplement: Supplementary file 1 [file ac5c00410_si_001.pdf]

Supporting Information

Exploring biochemical characteristics of pediatric hyperdiploid acute lymphoblastic leukemia by Raman spectroscopy

Anna M. Nowakowska[1], Patrycja Leszczenko[1,2], Agata Pastorczak[4], Zuzanna Urbańska[3,4], Justyna Jakubowska[3], Marta Ząbczyńska[3], Wojciech Mlynarski[3], Malgorzata Baranska[1], Kinga Ostrowska[3*], Katarzyna Majzner[1]*

[1]Jagiellonian University, Faculty of Chemistry, Gronostajowa 2, 30-387, Krakow, Poland

[2]Jagiellonian University in Kraków, Doctoral School of Exact and Natural Sciences, Lojasiewicza 11, Krakow, Poland

[3]Medical University of Lodz, Department of Pediatrics, Oncology and Hematology, Czechoslowacka 4, 92-216, Lodz, Poland

[4]Department of Genetic Predisposition to Cancer, Medical University of Lodz, Czechoslowacka 4, 92-216 Lodz, Poland

* Correspondence to: K. Majzner (katarzyna.b.majzner@uj.edu.pl), K. Ostrowska (kinga.ostrowska@umed.lodz.pl)

Table of Content

PCA analysis of HD samples based on their karyotype (Figure S1) 2

O-PLS-R model of leukemic cells depending on the number of chromosomes (Figure S2) 3

PCA analysis of HD samples based on their karyotype (Figure S1)

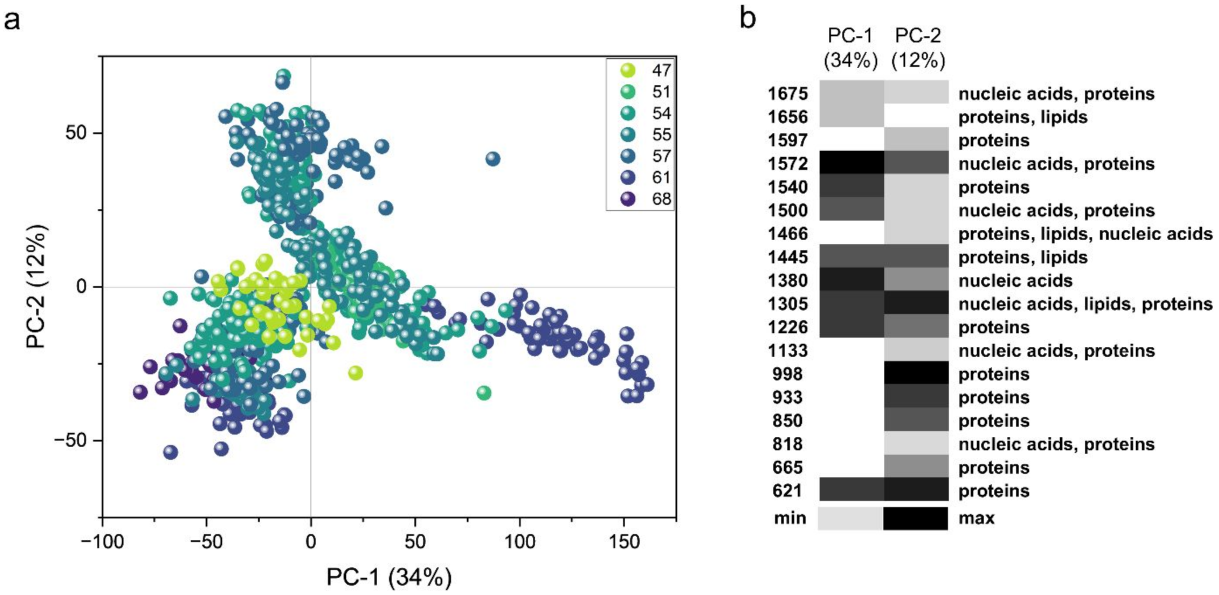

Figure S1. PCA analysis of HD samples based on their karyotype. (a) Two-dimensional score plots of PC-1 and PC-2. (b) Corresponding loading plots are presented in a color scale. Only bands for which the PC-1 and PC-2 had the highest values (PC-1: > 0.06 and < -0.06, PC-2: > 0.04 and < -0.04) were included. Analysis was carried out in the spectral range of 1800-610 cm^{-1}.

O-PLS-R model of leukemic cells depending on the number of chromosomes (Figure S2)

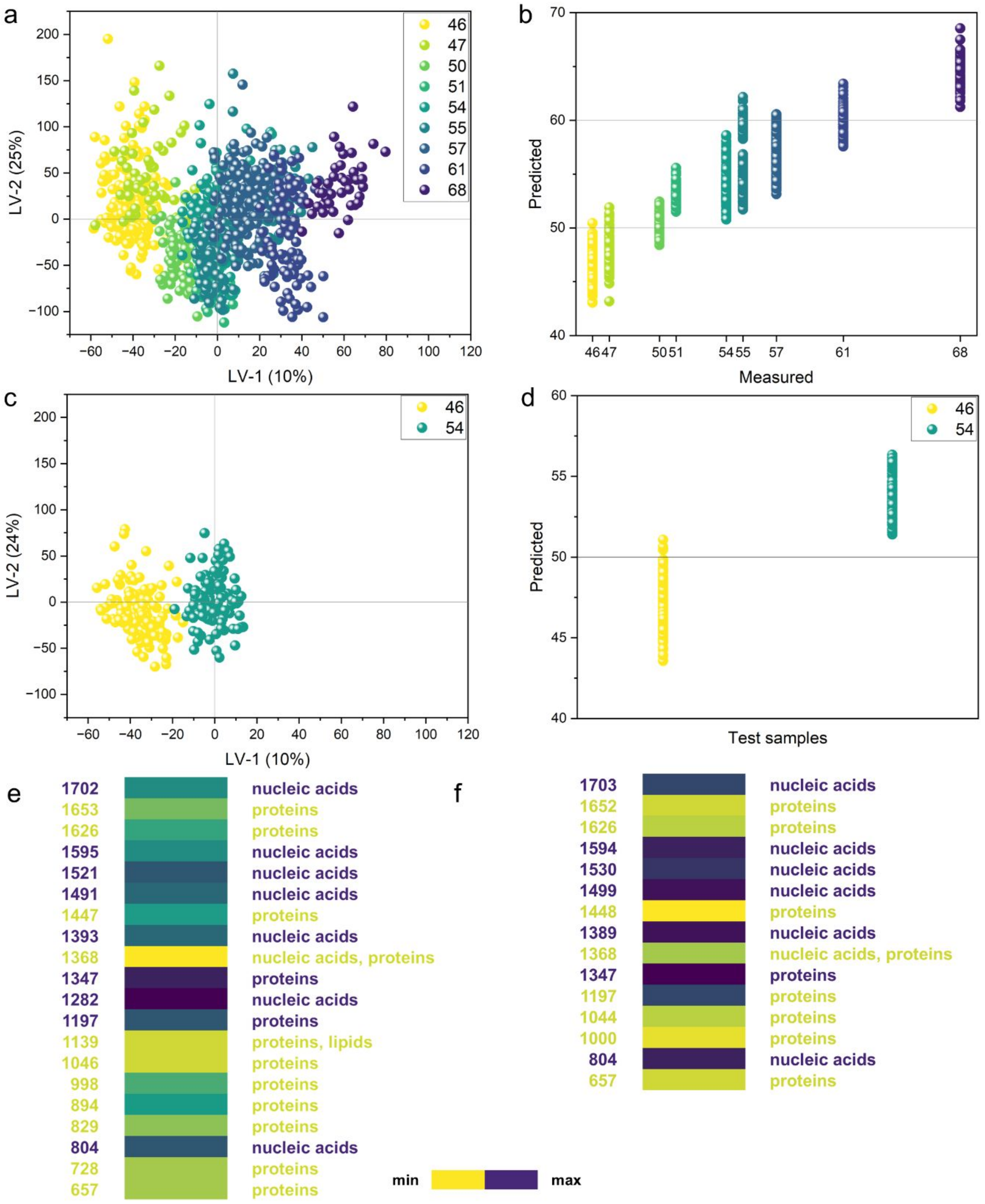

Figure S2. O-PLS-R model that examines the relationship between the Raman signal and the number of chromosomes in clinical samples calculated on the spectra of nuclei. (a) Score plot of the latent variables LV-1 and LV-2 for the training data set. In total, six LVs were used. (b) Model calibration result. (c) Score plot of the latent variables LV-1 and LV-2 for the test data set, which was not included in model training. (d) Prediction results of the model in test samples. (e) A plot of the regression vector of the model is presented on a color scale. Only bands for which the variable importance in projection (VIP) scores had the highest values (> 1) were included. (f) A plot of the LV-1 loading of the model is presented on a color scale. Only bands for which the LV-1 loading had the highest values (> 0.06 and < -0.06) were included. O-PLS-R analysis was performed in the spectral range of 1800 - 600 cm^{-1}.
